# Validation of a Deep Learning Tool in the Detection of Intracranial Hemorrhage and Large Vessel Occlusion

**DOI:** 10.3389/fneur.2021.656112

**Published:** 2021-04-29

**Authors:** Joel McLouth, Sebastian Elstrott, Yasmina Chaibi, Sarah Quenet, Peter D. Chang, Daniel S. Chow, Jennifer E. Soun

**Affiliations:** ^1^Department of Radiological Sciences, University of California, Irvine, Irvine, CA, United States; ^2^Avicenna.AI, La Ciotat, France; ^3^Center for Artificial Intelligence in Diagnostic Medicine, University of California, Irvine, Irvine, CA, United States

**Keywords:** deep learning, artificial intelligence, radiology, large vessel occlusion, neuroradiology, intracranial hemorrhage

## Abstract

**Purpose:** Recently developed machine-learning algorithms have demonstrated strong performance in the detection of intracranial hemorrhage (ICH) and large vessel occlusion (LVO). However, their generalizability is often limited by geographic bias of studies. The aim of this study was to validate a commercially available deep learning-based tool in the detection of both ICH and LVO across multiple hospital sites and vendors throughout the U.S.

**Materials and Methods:** This was a retrospective and multicenter study using anonymized data from two institutions. Eight hundred fourteen non-contrast CT cases and 378 CT angiography cases were analyzed to evaluate ICH and LVO, respectively. The tool's ability to detect and quantify ICH, LVO, and their various subtypes was assessed among multiple CT vendors and hospitals across the United States. Ground truth was based off imaging interpretations from two board-certified neuroradiologists.

**Results:** There were 255 positive and 559 negative ICH cases. Accuracy was 95.6%, sensitivity was 91.4%, and specificity was 97.5% for the ICH tool. ICH was further stratified into the following subtypes: intraparenchymal, intraventricular, epidural/subdural, and subarachnoid with true positive rates of 92.9, 100, 94.3, and 89.9%, respectively. ICH true positive rates by volume [small (<5 mL), medium (5–25 mL), and large (>25 mL)] were 71.8, 100, and 100%, respectively. There were 156 positive and 222 negative LVO cases. The LVO tool demonstrated an accuracy of 98.1%, sensitivity of 98.1%, and specificity of 98.2%. A subset of 55 randomly selected cases were also assessed for LVO detection at various sites, including the distal internal carotid artery, middle cerebral artery M1 segment, proximal middle cerebral artery M2 segment, and distal middle cerebral artery M2 segment with an accuracy of 97.0%, sensitivity of 94.3%, and specificity of 97.4%.

**Conclusion:** Deep learning tools can be effective in the detection of both ICH and LVO across a wide variety of hospital systems. While some limitations were identified, specifically in the detection of small ICH and distal M2 occlusion, this study highlights a deep learning tool that can assist radiologists in the detection of emergent findings in a variety of practice settings.

## Introduction

Timely diagnosis of acute cerebrovascular disease is critical to reduce patient mortality and morbidity. Two forms of stroke, intracranial hemorrhage (ICH) and ischemic stroke due to large vessel occlusion (LVO) are especially devastating. ICH 28-day mortality has been reported at 50.6% and 6-month mortality due to LVO at 26.2% (1, 2).

Prompt intervention of these entities is critical in achieving improved outcomes. For example, ICH hematoma expansion was significantly reduced with early blood pressure control (3). Regarding LVO, functional independence decreased with every hour delay to endovascular thrombectomy (4).

Deep learning, a subset of artificial intelligence, has recently emerged as a means to aid clinicians in the timely diagnosis of both ICH and LVO. Newly developed algorithms have demonstrated strong performance in the detection of each (5–12). However, limitations of most of these studies are that they are often performed at a single institution and have not been validated in different settings.

Given the potential for deep learning tools to aid physicians in the timely and accurate diagnosis of these emergencies, it is important to validate their uses across a variety of facilities. Prior studies examining the relationship between deep-learning based algorithms and imaging assessment have been limited by geographic bias introduced from their cohorts, with the majority of U.S. states lacking representation (13). The specific aim of this study is to validate a commercially available deep learning-based tool, CINA® v1.0 device (Avicenna.ai, La Ciotat, France) in the detection of both ICH and LVO from multiple hospital sites and vendors through a collaboration between the University of California, Irvine (UCI) and vRAD (Minneapolis, USA). In doing so, the goal was to evaluate the generalizability of this tool to eliminate possible geographic bias introduced in other similar studies.

## Materials and Methods

This was a retrospective study using anonymized data from UCI and vRAD. A waiver of consent was obtained from the local Institutional Review Board (IRB) at UCI for the UCI cases and the Western IRB for the vRAD cases. The CINA® v1.0 device (Avicenna.ai, La Ciotat, France) was used for standalone performance assessment in both the ICH and LVO validation studies. The statistics provided in this manuscript are derived from an external test set (the validation cohort) and are completely independent from a prior cohort used to train the CINA® v1.0 device. Specifically, the cohort used to train the tool was based off of 8,994 ICH cases acquired between November 2014–May 2018 and 566 LVO cases acquired between May 2018–November 2018. All of the training data was acquired from vRAD data only. No UCI data was used for the training cohort. Additionally, all vRAD cases used for the validation cohort were acquired in 2019 only.

### Intracranial Hemorrhage

#### Patient Selection

A cohort of patients with suspected acute ICH on clinical grounds in whom non-contrast CT (NCCT) head studies had been performed from UCI and an American teleradiology service (vRAD) were assessed. In both UCI and vRAD cases, suspected acute ICH cases were identified with keywords such as “hemorrhage,” “NCCT,” and “head” in the clinical indication or Digital Imaging and Communications in Medicine (DICOM) header information of the NCCT studies. Only the initial scan obtained for ICH evaluation was assessed for patients in this validation cohort. vRAD cases were acquired in 2019 only, and UCI data from 2017 to 2019. Inclusion criteria for NCCT scans required a strict axial acquisition, 512 x 512 matrix, slice thickness of <5 mm, soft tissue reconstruction kernel, and kVp ranging between 100 and 160.

ICH cases were divided into intraparenchymal (IPH), intraventricular (IVH), subarachnoid (SAH), subdural (SDH), and epidural (EDH) subtypes. Multiple cases contained a combination of these subtypes and were categorized accordingly. Intracranial hemorrhages were further categorized into small (<5mL), medium (5–25 mL), and large (>25 mL) volumes. Positive cases for acute ICH (“ground truths”) were assessed by two board-certified neuroradiologists, with consensus determined by a third board-certified neuroradiologist. The two board-certified neuroradiologists also determined ICH subtype and volume information.

#### Scanning Parameters

GE Healthcare, Philips, Siemens, and Canon (Formerly Toshiba) scanners were used among this cohort with 16, 7, 13, and 5 various scanner models, respectively. The number of detector rows (NDR) were divided into eight categories: 4 < NDR ≤ 8, 8 < NDR ≤ 16, 16 < NDR ≤ 32, 32 < NDR ≤ 64, 64 < NDR ≤ 128, 128 < NDR ≤ 256, 256 < NDR ≤ 320, and not available (NA) if this information was not attached to the case. Slice thickness (ST) was categorized as being <2.5 and 2.5 mm ≤ ST ≤ 5 mm. Radiation dose parameters were measured in kilovoltage peak (kVp) and milliampere-seconds (mAs). kVp was categorized as kVp <120, 120 ≤ kVp ≤ 140, and >140. mAs was categorized as <150, 150 ≤ mAs ≤ 400, and >400.

### Large Vessel Occlusion

#### Patient Selection

A cohort of patients with suspected LVO on clinical grounds in whom CT angiography (CTA) head studies had been performed from UCI and vRAD were assessed. For both UCI and vRAD cases, suspected LVO cases were identified with keywords such as, “CTA,” “head,” and “large vessel occlusion” in the clinical indication or DICOM header information of the CTA studies. Only the initial scan obtained for LVO evaluation was assessed for patients in this validation cohort. vRAD cases were acquired in 2019 only and UCI cases from 2015 to 2019. Inclusion criteria for CTA scans included a strict axial acquisition, 512 x 512 matrix, slice thickness ≤ 1.25 mm, kVp to range between 80 and 140, arterial phase timing of contrast bolus confirmed by mini test bolus or automatic bolus tracking software, and arterial (or other sharp) reconstruction kernel. Positive cases for LVO (“ground truths”) were assessed by two U.S. board-certified neuroradiologists, with consensus determined by a third board-certified neuroradiologist. Positive LVO cases were divided based on location into Distal Internal Carotid Artery (ICA), Middle Cerebral Artery (MCA)-M1, MCA-M2 Proximal, and MCA-M2 Distal.

#### Scanning Parameters

GE Medical Systems, Philips, Siemens, Canon (Formerly Toshiba), and NMS with 13, 4, 12, 4, and 1 various scanner models were included, respectively. The NDR were divided into the same eight categories as for the ICH cases. Slice thickness (ST) was ≤ 1.25 mm. Radiation dose parameters were measured in kilovoltage peak (kVp) and milliampere-seconds (mAs). kVp was categorized as <100, 100 ≤ kVp ≤ 120, and >120. mAs were categorized as <100, 100 ≤ mAs ≤ 400, and >400.

### Statistical Analysis

Data was compared from the CINA® v1.0 device (Avicenna.ai, La Ciotat, France) to the ground truths determined by the board-certified neuroradiologists via a confusion matrix in order to obtain sensitivity, specificity, and accuracy. Positive predictive values (PPV) and negative predictive values (NPV) were computed with varying prevalence values (from 10 to 50%, increments of 5%). All of these statistics were performed using Excel and MedCalc version 19.7.2.

These statistics were performed for the total cases in both the ICH and LVO groups in addition to stratifications based on scanner models, NDR, slice thickness, radiation dose parameters, age, and sex, as well as ICH subtypes and volumes and LVO locations. The CINA® v1.0 device is not intended to discern ICH subtype or volume and only detects whether hemorrhage is present or not. Therefore, ICH subtypes and volume information were only assessed in positive cases by the two board-certified neuroradiologists. Only true positives and false negative values could be calculated and only true positive rate was provided for these classifications.

PASS sample size software was used to calculate the minimum number of cases needed to achieve a 95% CI lower bound of at least 80% assuming a point estimate of 90% (for sensitivity and specificity, separately). Using the binomial dichotomous endpoint for a one sample study, at least 137 positive and 137 negative anonymized cases were required (for ICH and LVO).

## Results

### Intracranial Hemorrhage

#### Patient Selection

824 cases were selected for analysis from a pool of 400 retrospective anonymized cases from vRAD and 424 from UCI. 10 cases were excluded for the following reasons: 1 because slice thickness was not identical among the volume, 3 because the matrix was not 512 x 512, 1 because it contained a post-contrast series, 2 lacked a full field of view, 2 were uninterpretable due to significant motion artifact, and 1 was uninterpretable due to significant metal artifact. After exclusion, case distribution was 395 from vRAD and 419 from UCI for a total of 814 cases.

#### Overall Cases

ICH ground-truths were as follows: 204 positive ICH cases from vRAD and 51 from UCI. There were 191 negative ICH cases from vRAD and 368 from UCI. There was initial disagreement on 21 cases between the two neuroradiologists. However, a consensus was eventually reached for each of these cases.

**Figure 1:**
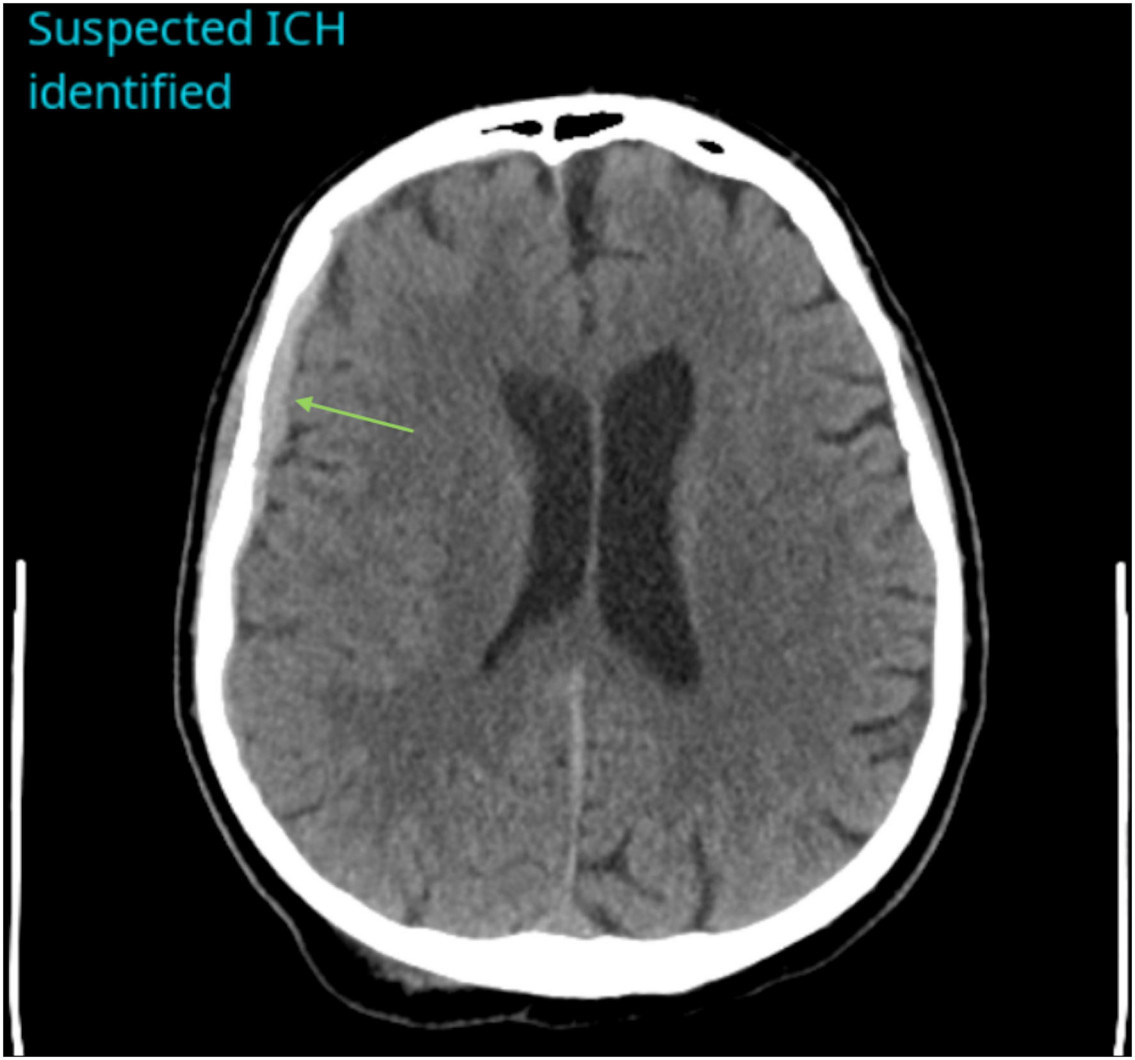
Example of a positive ICH identified by CINA®. There is an acute subdural hemorrhage along the right cerebral convexity on non-contrast CT (green arrow).

The CINA® v1.0 algorithm identified 233 true positive ICH (Figure 1), 14 false positive ICH, 545 true negative ICH, and 22 false negative ICH. Accuracy was calculated as 95.6%. Sensitivity was 91.4 % [95% CI, 87.2–94.5%] and specificity was 97.5% [95% CI, 95.8–98.6%]. Performance metrics can be found in Table 1.

**Table 1:** Performance metrics for overall cases of CINA-ICH application.

**Statistical findings for ICH detection**	**Values [95% CI]**
Sensitivity (%) [95% CI]	91.4% [87.2–94.5%]
Specificity (%) [95% CI]	97.5% [95.8–98.6%]

Positive predictive values (PPV) and negative predictive values (NPV) were also assessed based on varying prevalence. PPV ranged from 80.2% (10% prevalence) to 97.3% (50% prevalence). NPV ranged from 99.0% (10% prevalence) to 91.9 % (50% prevalence).

#### Demographics

Performance metrics for age and sex can be found in Table 2. For age, sensitivity ranged from 89.2% (18 ≤ Age <40, *n* = 168) to 92.9% (Age > 70; *n* = 249). Specificity ranged from 96.1% (40 ≤ Age ≤ 70, n=316) to 100% (18 ≤ Age <40, n=168). For sex, sensitivity was 91.4% for females (*n* = 188) and 93.6% for males (*n* = 206). Specificity was 97.9% for females and 96.8% for males.

**Table 2:** Performance metrics for ICH cases based on demographics.

**Demographic**		**Positive ICH**	**Negative ICH**	**Sensitivity (%)**	**Specificity (%)**
Age	18 ≤ Age < 40 (*n* = 168)	37	131	89.2	100
	40 ≤ Age ≤ 70 (*n* = 316)	112	204	91.1	96.1
	Age > 70 (*n* = 249)	98	151	92.9	97.4
	Age NA (*n* = 81)	8	73	–	–
Sex	Male (*n* = 206)	101	95	93.6	96.8
	Female (*n* = 188)	93	95	91.4	97.9
	NA (*n* = 421)	52	359	–	–

*NA, Not Available*.

#### Site

The distribution, sensitivity, and specificity of the ICH tool based on geographic U.S. regions are shown in Table 3. Sensitivity ranged from 88% (Southeast U.S., *n* = 96) to 93.1% (Continental U.S., *n* = 55). Specificity ranged from 95.2% (Northeast U.S., *n* =187) to 100% (Continental and Southeast U.S.).

**Table 3:** Performance metrics for ICH cases by geographic distribution in the United States.

**Region category**	**Positive ICH**	**Negative ICH**	**Sensitivity (%)**	**Specificity (%)**
Continental (*n* = 55)	29	26	93.1	100
Northeast (*n* = 187)	83	104	91.6	95.2
Pacific (*n* = 450)	67	383	89.5	97.6
Southeast (*n* = 96)	50	46	88	100
NA (*n* = 26)	26	0	–	–

*NA, not available*.

#### ICH Subtypes

ICH cases were additionally categorized based on subtypes: Intraparenchymal (IPH), Intraventricular (IVH), Subarachnoid (SAH), Subdural (SDH), and Epidural (EDH). The SDH and EDH subtypes were combined into one group for stratification purposes. In addition, some patients are represented across multiple categories (IPH, IVH, EDH/SDH, and SAH). Distribution is seen in Table 4. CINA® demonstrated a true positive rate of 92.9% for IPH, 100% for IVH, 94.3% for EDH/SDH, and 89.9% for SAH. ICH size distribution is also seen in Table 4. CINA® demonstrated a true positive rate of 71.8% for small (<5 mL) ICH, 100% for medium (5–25 mL), and 100% for large (>25 mL) ICH.

**Table 4:** True positive rates for ICH cases based on subtype and volume.

**ICH classification**		**True positive rate (%)**
Subtype	IPH (*n* = 99)	92.9
	IVH (*n* = 23)	100
	EDH/SDH (*n* = 122)	94.3
	SAH (*n* = 79)	89.9
Volume	Small: <5 mL (*n* = 78)	71.8
	Medium: 5–25 mL (*n* = 100)	100
	Large: >25 mL (*n* = 77)	100

#### Scanner Models

Case distribution among the various scanner models are found in Table 5. Note that there were 41 different scanner models for the ICH data set with 16, 7, 13, and 5 different models for GE, Philips, Siemens, and Canon (Formerly Toshiba), respectively. Sensitivity for GE, Philips, Siemens, and Canon was 91.8, 84.4, 96.7, and 94.1%, respectively. Specificity was 98.1, 97.7, 90.0, and 100%, respectively.

**Table 5:** Performance metrics and distribution for ICH cases based on different scanning parameters.

**Scanning parameter**		**Positive ICH**	**Negative ICH**	**Sensitivity (%)**	**Specificity (%)**
Scanner model	GE Healthcare (*n* = 203)	97	106	91.8	98.1
	Philips (*n* = 461)	64	397	84.4	97.7
	Siemens (*n* =90)	60	30	96.7	90
	Canon (Formerly Toshiba) (*n* = 60)	34	26	94.1	100
Detector rows	4 < NDR ≤ 8 (*n* = 2)	2	0	100	–
	8 < NDR ≤ 16 (*n* = 46)	14	32	78.6	100
	16 < NDR ≤ 32 (*n* = 185)	91	94	94.5	96.8
	32 < NDR ≤ 64 (*n* = 518)	111	407	91	97.5
	64 < NDR ≤ 128 (*n* = 6)	5	1	100	100
	128 < NDR ≤ 256 (*n* = 12)	8	4	87.5	100
	256 < NDR ≤ 320 (*n* = 14)	12	2	100	100
	NA (*n* = 31)	12	19	–	–
Slice Thickness	ST <2.5 mm (*n* = 39)	23	16	100	100
	2.5 ≤ ST ≤ 5 mm (*n* = 775)	232	543	90.5	97.4
kVp	kVp <120 (*n* = 8)	6	2	100	100
	120 ≤ kVp ≤ 140 (*n* = 806)	249	557	91.2	97.5
	kVp >140 (*n* = 0)	0	0	–	–
mAs	mAs <150 (*n* = 23)	12	11	100	90.9
	150 ≤ mAs ≤ 400 (*n* = 765)	231	534	90.9	97.8
	mAs > 400 (*n* = 26)	12	14	91.7	92.9

*NA, not available*.

#### Scanning Parameters

Case distribution and performance metrics among the various scanning parameters can be found in Table 5. The number of detector rows' sensitivity ranged from 78.6% (8 < NDR ≤ 16, *n* = 46) to 100% (4 < NDR ≤ 8, *n* = 2; 64 < NDR ≤ 128, *n* = 6; 256 < NDR ≤ 320, *n* = 14). Specificity ranged from 96.8% (16 < NDR ≤ 32, *n* = 185) to 100% (8 < NDR ≤ 16; 64 < NDR ≤ 128; 128 < NDR ≤ 256, *n* = 12; 256 < NDR ≤ 320). Slice thickness sensitivity and specificity was 100% when slice thickness was <2.5 mm. Sensitivity was 90.5% and specificity was 97.4% when slice thickness was 2.5 ≤ ST ≤ 5 mm. Sensitivity and specificity for kilovoltage peaks was 100% when kVp was <120. Sensitivity was 91.2% and specificity was 97.5% when kVp was 120 ≤ kVp ≤ 140. Sensitivity for milliampere-seconds was 100% and specificity was 90.9% when mAs <150. Sensitivity was 90.9% and specificity was 97.8% when 150 ≤ mAs ≤ 400. Sensitivity was 91.7% and specificity was 92.9% when mAs was >400.

### Large Vessel Occlusion

#### Patient Selection

406 anonymized CT angiography (CTA) cases were assessed; 93 from UCI and 313 from vRAD. 28 of these were excluded for the following reasons: 11 were not CTAs, 2 had no contrast, 7 did not have enough contrast, 2 were uninterpretable due to significant motion artifact, 2 were uninterpretable due to significant metal artifact, 3 did not have a full field of view, and 1 had an acquisition issue (z-spacing variability).

#### Overall Cases

LVO ground-truths (determined by two board-certified neuroradiologists) were as follows: 156 positive LVO cases and 222 negative LVO cases. There was initial disagreement on 19 cases between the two neuroradiologists. However, a consensus was eventually reached for each of these cases.

**Figure 2:**
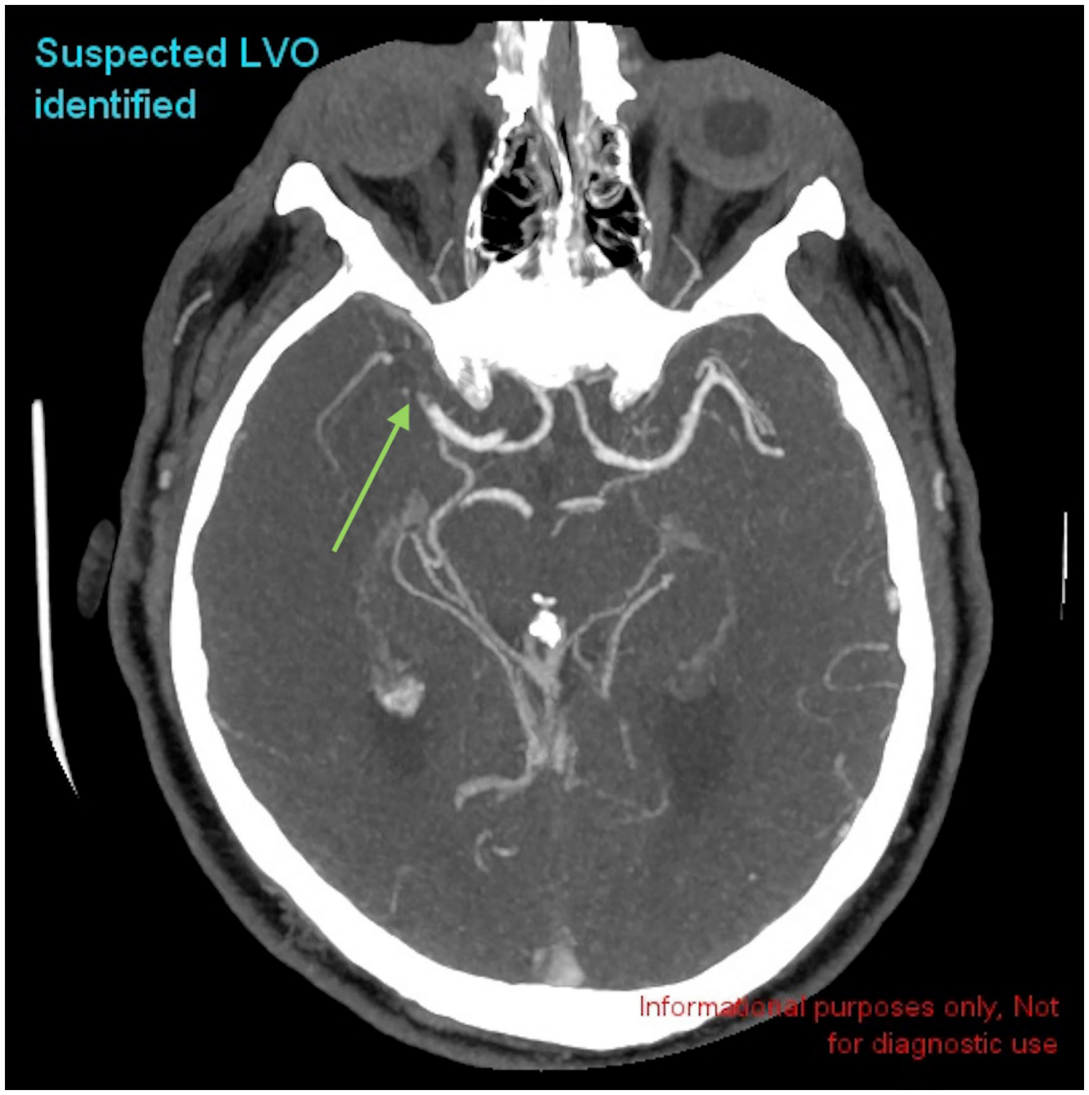
Example of a positive LVO identified by CINA®. There is a large vessel occlusion in the distal right MCA-M1 branch on CTA (green arrow).

The CINA® v1.0 algorithm identified 153 true positive LVO (Figure 2), 4 false positive LVO, 218 true negative LVO, and 3 false negative LVO. Sensitivity was 98.1 % [95% CI, 94.0–99.5%] and specificity was 98.2% [95% CI, 95.1–99.4%]. Performance metrics can be found in Table 6.

**Table 6:** Performance metrics for overall cases of CINA-LVO application.

**Statistical findings for LVO detection**	**Values [95% CI]**
Sensitivity (%) [95% CI]	98.1% [94–99.5%]
Specificity (%) [95% CI]	98.2% [95.1–99.4%]

Positive predictive values (PPV) and negative predictive values (NPV) were also assessed based on varying prevalence (from 10 to 50%, increments of 5%). PPV ranged from 85.8% (10% prevalence) to 98.2% (50% prevalence). NPV ranged from 99.8% (10% prevalence) to 98.1% (50% prevalence).

#### Demographics

Performance metrics for patient demographics can be found in Table 7. For age, sensitivity ranged from 83.3% (18 ≤ Age <40, *n* = 26) to 100% (40 ≤ Age ≤ 70, *n* = 176). Specificity ranged from 97.3% (40 ≤ Age ≤ 70) to 100% (18 ≤ Age <40). For sex, sensitivity was 97.5% for females (*n* = 186) and 98.7% for males (*n* = 185). Specificity was 98.1% for females and 98.2% for males.

**Table 7:** Performance metrics for LVO cases among different demographic parameters.

**Demographic**		**Positive LVO**	**Negative LVO**	**Sensitivity (%)**	**Specificity (%)**
Age	18 ≤ Age < 40 (*n* = 26)	5	21	83.3	100
	40 ≤ Age ≤ 70 (*n* = 176)	65	111	100	97.3
	Age > 70 (*n* = 176)	85	91	97.7	98.9
Sex	Male (*n* = 185)	74	111	98.7	98.2
	Female (*n* = 186)	80	106	97.5	98.1
	NA (*n* = 7)	2	5	–	–

*NA, not available*.

#### Site

The distribution, sensitivity, and specificity of the LVO tool based on geographic U.S. regions are shown in Table 8. Sensitivity ranged from 97.4% (Southeast U.S., *n* = 75) to 100% (Continental U.S., *n* = 27). Specificity ranged from 97.3% (Southeast U.S.) to 100% (Continental U.S.).

**Table 8:** Performance metrics for LVO cases by geographic distributions in the United States.

**Region category**	**Positive LVO**	**Negative LVO**	**Sensitivity (%)**	**Specificity (%)**
Continental (*n* = 27)	8	19	100	100
Northeast (*n* = 155)	50	105	98	98.1
Pacific (*n* = 120)	59	61	98.3	98.4
Southeast (*n* = 75)	38	37	97.4	97.3
NA (*n* = 1)	1	0	–	–

*NA, not available*.

#### LVO Subtypes

A subset of 55 patients were randomly selected to evaluate performance metrics of the tool in evaluating LVO subtypes (4 Distal ICA, 26 MCA-M1, 20 Proximal MCA-M2, and 3 Distal MCA-M2). Accuracy was 97.0%, sensitivity 94.3% [95% CI, 83.4–98.5%], and specificity 97.4% [95% CI, 95.1–98.7%].

#### Scanner Models

Case distribution among the various scanner models can be found in Table 9. Sensitivity for Siemens, Canon (formerly Toshiba), GE Medical Systems and Philips was 96.7, 94.1, 91.8, and 84.4%, respectively. Specificity for GE Healthcare, Philips, Siemens, Canon, and NMS was 90.0, 100, 98.1, 97.7, and 100%, respectively.

**Table 9:** Performance metrics for LVO cases based on different scanning parameters.

**Scanning parameter**		**Positive LVO**	**Negative LVO**	**Sensitivity (%)**	**Specificity (%)**
Scanner model	GE healthcare (*n* = 129)	50	79	91.8	98.1
	Philips (*n* = 137)	62	75	84.4	97.7
	Siemens (*n* = 73)	30	43	96.7	90.0
	Canon (Formerly Toshiba) (*n* = 37)	14	23	94.1	100
	NMS (*n* = 2)	0	2	–	100
Detector rows	4 < NDR ≤ 8 (*n* = 0)	0	0	–	–
	8 < NDR ≤ 16 (*n* = 15)	5	10	100	90
	16 < NDR ≤ 32 (*n* = 63)	27	36	100	100
	32 < NDR ≤ 64 (*n* = 146)	52	94	96.2	96.8
	64 < NDR ≤ 128 (*n* = 126)	65	61	98.5	100
	128 < NDR ≤ 256 (*n* = 0)	0	0	–	–
	256 < NDR ≤ 320 (*n* = 5)	1	4	100	100
	NA (n=23)	6	17	–	–
Slice thickness	ST ≤ 1.25 mm (*n* = 378)	156	222	98.1	98.2
kVp	kVp <100 (*n* = 8)	1	7	100	100
	100 ≤ kVp ≤ 120 (*n* = 359)	150	209	98	98.6
	kVp > 120 (*n* = 11)	5	6	100	83.3
mAs	mAs <100 (*n* = 43)	11	32	100	100
	100 ≤ mAs ≤ 400 (*n* = 314)	138	176	97.8	97.7
	mAs > 400 (*n* = 21)	7	14	100	100

*NA, not available*.

#### Scanning Parameters

Case distribution and performance metrics among the various scanning parameters can be found in Table 9. Sensitivity for the number of detector rows ranged from 96.2% (32 < NDR ≤ 64, *n* = 146) to 100% (8 < NDR ≤ 16, *n* = 15; 16 < NDR ≤ 32, *n* = 63; 256 < NDR ≤ 320, *n* = 5). Specificity ranged from 90.0% (8 < NDR ≤ 16) to 100% (16 < NDR ≤ 32; 64 < NDR ≤ 128, *n* = 126; 256 < NDR ≤ 320). Sensitivity and specificity for slice thickness ≤ 1.25 mm was 98.1 and 98.2%, respectively. Sensitivity for kilovoltage peak ranged from 98% (100 ≤ kVp ≤ 120, *n* = 359) to 100% (kVp <100, *n* = 8; kVp > 120, *n* = 11). Specificity ranged from 83.3% (kVp > 120) to 100% (kVp <100). Sensitivity for milliampere-seconds ranged from 97.8% (100 ≤ mAs ≤ 400, *n* =314) to 100% (mAs <100, n=43; mAs >400, *n* = 21). Specificity ranged from 97.7% (100 ≤ mAs ≤ 400) to 100% (mAs <100; mAs > 400).

## Discussion

This retrospective, multicenter study aimed to demonstrate the generalizability of a commercially available deep-learning based tool, CINA® v1.0, in the detection of ICH and LVO across multiple hospital settings. The algorithm performed well in the ICH cohort, with an overall accuracy of 95.6%, sensitivity of 91.4%, and specificity of 97.5%. Of the ICH subtypes, it achieved the highest sensitivity in the detection of intraventricular hemorrhage with a true positive rate of 100%, followed by epidural/subdural, intraparenchymal, and subarachnoid subtypes which all had sensitivity of at least 90%. When stratified by ICH size, it performed best for medium and large volumes with sensitivities of 100%, but demonstrated lower sensitivity in the detection of small volumes with a sensitivity of 71.8%.

The tool also performed well in the LVO cohort, with an accuracy of 98.1%, sensitivity of 98.1%, and specificity of 98.2%. The algorithm showed robust performance in detecting LVO location in a smaller subset of cases with an accuracy of 97.0%, sensitivity of 94.3%, and specificity of 97.4%.

These results corroborate previous studies analyzing the ability for deep-learning tools to detect intracranial emergencies. For example, Chilamkurthy et al. used a deep-learning algorithm to detect and classify ICH on large and diverse cohorts in India (6). Another study obtained an AUC of 0.99 in the detection of ICH via deep-learning algorithms; however, this was based off of a single institution using relatively uniform scanning parameters on two scanner models (9). Similar studies have been performed with respect to AI detection of LVO. For example, a commercially available deep learning software for LVO detection achieved an AUC of 0.86 using a cohort derived from three tertiary stroke centers (11). Our work expands on these previous studies by showing similar robust performance of deep learning tools across a diverse population regardless of scanner parameters and geographic distribution.

Ultimately, given the robust nature of deep learning tools such as CINA® v1.0, the goal of these tools is to streamline the radiologists' workflow by triaging studies to alert physicians to the most time-sensitive findings, and to act as a second set of eyes when studies are more ambiguous. Studies evaluating the effectiveness of such systems have already begun. For example, when a deep-learning tool was prospectively integrated to prioritize studies in a radiologist's workflow based on the presence of ICH, one study found that time to diagnosis was significantly reduced (14). Future studies with CINA® v1.0 could mirror this type of work and evaluate patient outcomes as influenced by the integration of deep-learning tools into a radiologist's workflow. For example, both inpatient and outpatient settings could be evaluated with regards to these neurologic emergencies and how these tools impact efficiency and ultimately clinical outcomes.

Our software had some limitations that warrant further investigation. Perhaps the greatest limitation was in the detection of small bleeds, with false negatives occurring predominantly in very small ICH (<1.5 mL). The false negatives that occurred in larger bleeds (1.5–5 mL), were often located within chronic pathology such as old hematomas, areas of gliosis, or extra-parenchymal structures such as along the falx cerebri. On the other hand, ICH false positives predominantly occurred in the setting of significant streak or motion artifact. Similar limitations were identified in the LVO cohort with respect to imaging artifact and small size. For example, the LVO false negatives all occurred in the setting of small occlusions <1.3 mm in length (Note that LVO lengths were only retrospectively measured for the three false negative cases in order to understand why the application failed to detect them and were not measured for the remaining LVO cases). A false positive LVO case also occurred in the setting of significant streak artifact. Another false positive case misdiagnosed an area of stenosis as a complete occlusion. Two false positive cases misidentified the sphenoparietal sinus venous structure as an area of occlusion, likely secondary to its close proximity to the MCA. As a result, caution should be used when relying solely on the software in these settings. However, the limitations discussed above often occurred in settings that would likely pose similar challenges to radiologists and result in a similar distribution of false negatives and positives.

CINA® v1.0 was only trained to identify acute blood based off of hyperdense components. Thus, chronic hemorrhages cannot be identified by the algorithm unless they contain more acute hyperdense components. However, given that low density hemorrhages (e.g., chronic SDH) are often not emergencies, we believe this distinction is actually clinically useful and not necessarily a limitation in order to prevent the tool from flooding the radiologist with alerts for non-emergent cases. The tool did not differentiate between acute and non-acute LVO etiologies such as chronic ICA occlusion, and these may have been included as positive LVO cases. Lastly, the tool was not trained to evaluate occlusions in the anterior cerebral arteries or posterior circulation. While further work is needed for future tools to better identify more distal occlusions and subtle hemorrhages, the primary goal of CINA in its current state is to identify obvious findings that need to be assessed urgently for emergent triage and prioritization of the worklist. This is reflected in a separate standalone effectiveness assessment demonstrating a mean “time-to-notification” of 21.6 and 34.7 s for ICH and LVO detection, respectively.

Despite these limitations, CINA® v1.0 demonstrates robust generalizability in the detection of ICH and LVO. For example, in a study examining the geographic distribution of various cohorts evaluated by deep learning based algorithms in various medical specialties, 34 states were not represented among 56 studies (13). Our ICH data spans 44 states and 204 U.S. cities, while LVO data reflects scans from 40 states and 158 U.S. cities. To our knowledge, this is the most heterogeneous population cohort ever studied in the U.S. using a deep learning tool for ICH and LVO detection. This study demonstrates the potential for greater application of deep-learning tools across a wide variety of clinical settings.

## Data Availability Statement

The raw data supporting the conclusions of this article will be made available by the authors, without undue reservation.

## Ethics Statement

The studies involving human participants were reviewed and approved by University of California, Irvine Institutional Review Board, and Western Institutional Review Board. Written informed consent for participation was not required for this study in accordance with the national legislation and the institutional requirements.

## Author Contributions

JM and SE: manuscript preparation, data gathering, analysis. YC: manuscript revision, data gathering and analysis. SQ: data gathering and analysis. PC: data analysis, software programming. DC: data analysis. JS: manuscript revision, study design, data gathering and analysis. All authors contributed to the article and approved the submitted version.

## Conflict of Interest

YC and SQ are employees of Avicenna.ai. PC is co-founder of and owns stock in Avicenna.ai, has past and current research funding from and is a paid consultant for Canon Medical, has current research funding from GE, and is a paid consultant and speaker for Siemens. DC has a grant with Avicenna.ai (money paid to institution), has done consultancy for Canon Medical, has done expert testimony for Cullins & Grandy, has grants/grants pending with Canon Medical and Novocure, and has stock/stock options with Avicenna.ai. The remaining authors declare that the research was conducted in the absence of any commercial or financial relationships that could be construed as a potential conflict of interest.
